# Anti-VEGF versus laser photocoagulation for ROP treatment

**DOI:** 10.6026/973206300221274

**Published:** 2026-02-28

**Authors:** Sanskriti Ukey, Shivam Pandey, Anjali Singh, Anamika Dwivedi, Sachin Parmar

**Affiliations:** 1 Department of Ophthalmology, NandKumar Singh Chauhan Medical college Khandwa, Madhya Pradesh, India; 2 Department of Ophthalmology, N. S. C. B. Medical College, Jabalpur, Madhya Pradesh, India; 3 Department of Ophthalmology, Chhindwara Institute of Medical Sciences and attached District Hospital, Chhindwara, Madhya Pradesh, India; 4 Department of Ophthalmology, Shyam Shah Medical College, Rewa, Madhya Pradesh, India; 5 Department of Community Medicine, V.K.S. Government Medical College, Neemuch, Madhya Pradesh, India

**Keywords:** Retinopathy of prematurity (ROP), anti-VEGF therapy, laser photocoagulation

## Abstract

Retinopathy of prematurity (ROP) is a vascular disease of the developing retina and a key, but mostly avoidable, cause of blindness in infants (especially preterm and very low birth weight infants). Hence in this prospective comparative cohort study of 1188 screened infants, 77 infants needed treatment; 67 of them received intravitreal anti-VEGF and 10 received laser photocoagulation. Intravitreal anti-VEGF has shown anatomical regression in 96.82 of treated eyes; of which 3.17% required auxiliary laser and primary laser anatomical success was 100% but ablate the peripheral retina more and theoretically presents a greater risk of myopic shift. Aggressive posterior ROP (APROP) which was observed in 5.5 percent of the cohort responded to anti -VEGF in most cases, although isolated but serious complications were identified including cataract and retinal detachment, which demonstrates the importance of careful long-term follow-up. Thus Anti-VEGF was also useful in Type 1 ROP, but the current study also makes progress in demonstrating in a clinical setting that anti-VEGF can yield high regression with low adjunctive laser and superior peripheral retinal sparing, as opposed to laser which has a little higher one-time anatvi-success, indicating that a customized, zone- and stage-based, risk-benefit-driven decision on which therapy to use in any infant is possible.

## Background:

Retinopathy of prematurity (ROP) is a vascular retinopathy of preterm infants and is one of the most common avoidable causes of childhood blindness [1]. The aberrant retinogenesis is caused by vascular endothelial growth factor (VEGF) in its pathogenesis and this has been central in the transition of early-vascular breakdown to later-pathological neovascularization [2]. The illness is most frequently affecting infants with a very low gestational time and birth weight; additionally, the level of anatomic location, illness phase and the presence of plus disease define the severity of the disease [3]. Type 1 ROP In ROP, laser photocoagulation proved to be the standard modality on which Early Treatment of Retinopathy of Prematurity (ETROP) trial found that ROP should be treated immediately, [4] providing evidence that ROP was a disease that should be treated as soon as it appeared [5]. Diode laser photocoagulation of the peripheral avascular retina helps the reduction of the disease by suppressing VEGF production and preventing neo-angiogenesis [6]. The procedure has shown anatomic success rates of more than 90% in the treated eyes where retinal detachment is avoided and the central vision is maintained. However, laser treatment has a number of side effects such as irreversible peripheral retinal damage, a visual field constriction and high likelihood of developing high myopia [7]. Introduction of intravitreal anti-VEGF agents has revolutionized the ROP therapy as it selectively suppresses angiogenic cascade underlying it [8]. The Bevacizumab Eliminates the Angiogenic Threat for Retinopathy of Prematurity (BEAT -ROP) trial was the original large, randomized trial supporting intravitreal bevacizumab and proving it to be more effective than its usual counterpart, laser photocoagulation, especially when the disease involves zone I and posterior zone II retina [9]. Other research has added ranibizumab and aflibercept to the list of therapeutic options; both have unique pharmacokinetics and clinical efficacy [10]. Recent meta-analyses and systematic reviews have given strong evidence concerning the relative effectiveness of these treatment modalities [11]. In a large meta-analysis study conducted using seven randomize controlled trials on 579 infants; the intravitreal anti-VEGF monotherapy showed that the adverse event was significantly lower with this method compared with laser photocoagulation. A similar pooled analysis showed that there were no statistically significant differences in the two methods in terms of recurrence rates, therapeutic switching and necessity to go back to treatment or mortality [12]. Conversely, zone-specific studies report that anti-VEGF treatment can be of specified advantages to zone 1 ROP with an improved visual acuity and minimum myopic power defect relative to laser treatment [13]. The RAINBOW trial, the first study that directly compared ranibizumab with laser photocoagulation, has made ranibizumab become the first anti-VEGF agent ever approved by the U.S. Food and Drug Administration in the treatment of ROP [10]. The trial established that ranibizumab 0.2mg dose resulted in an 80 per cent therapeutic response rate which was better than the 66 per cent response rate with laser therapy and with fewer structural complications. The extension study of RAINBOW was a long-term study that reinforced the already discovered reduction of high-myopia incidence after the administration of ranibizumab and maintained a similar level of visual-acuity results at five years of follow-up [14]. There are a number of conceptual benefits of anti-VEGF therapy over laser therapy, including the preservation of peripheral retinal tissue and the possibility of physiologic vascularization to continue to occur [15]. In addition to the aforementioned, recent studies have also report that both modalities lead to constricted visual fields when compared to normal controls [16]. However, visual fields in eyes treated with anti VEGF are wider than those in eyes treated with laser, which could translate into better functional outcomes. Nevertheless, there are still some concerns regarding anti-VEGF therapy [17]. A higher recurrence rate is the primary concern, as it necessitates more frequent retreatment than laser photocoagulation. The recurrence rate is reported to range between 20% and 60%, depending on the anti-VEGF agent used [18]. Therefore, it is of interest to show how different treatment modalities influence the balance between visual field preservation and recurrence risk in clinical practice.

## Materials and Methods:

The study titled "Comparing Intravitreal Anti-VEGF Agents and Laser Photocoagulation for ROP Treatment" was executed in the Department of Ophthalmology, Shyam Shah Medical College, Rewa (M.P.) from September 2022 to February 2024. This was a prospective comparative study conducted on all infants (both inborn and outborn) diagnosed with treatment-requiring retinopathy of prematurity subsequent to universal eye screening.

## Study design and location:

This was a hospital-based prospective comparative study designed to assess the efficacy and safety outcomes of intravitreal anti-VEGF agents compared to laser photocoagulation in the management of severe ROP.

## Criteria for inclusion: 

[1] Infants diagnosed with Type 1 ROP necessitating immediate intervention according to ETROP guidelines. - Zone I ROP: any stage exhibiting plus disease.

[2] Zone I ROP: stage 3 without any plus disease

[3] Zone II ROP: stage 2 or 3 with the plus disease

[4] Aggressive ROP (AROP)

[5] Babies with bad ROP presentation (Stage 4A, 4B or Stage 5)

[6] Born at 34 weeks or less of pregnancy - Weight at birth of 1500 grams or less

[7] Approval from parents for treatment and follow-up

## Criteria for exclusion:

[1] Newborns with severe ocular anomalies that hinder proper fundus examination (Microcornea, sclerocornea, corneal opacity, congenital cataract)

[2] Infants with significant systemic malformations impacting survival

[3] Cases with incomplete follow-up data - Parents refusing consent for treatment or study participation

## Assessment before treatment:

A pediatrician did a full clinical exam, recording the patient's age, weight at birth, number of pregnancies, mode of delivery and neonatal risk factors like respiratory distress syndrome, anemia, neonatal hyperbilirubinemia, sepsis, length of oxygen supplementation and need for phototherapy.

## Protocol for eye exams:

A full eye exam included looking at the outside of the eyes, checking the cornea's clarity, the pupils' reactions, the lens's status (tunica vasculosalentis) and a detailed fundus exam after the pupils were dilated.

## Dilation of the pupils:

Thirty minutes before the exam, topical mydriatic drops with 0.5% Tropicamide and 2.5% Phenylephrine hydrochloride were put both eyes every 5 to 10 minutes for 2 to 3 applications.

## Instrumentation:

We used a binocular indirect ophthalmoscope, a +20D aspheric lens, a pediatric eye speculum, a pediatric scleral depressor, a topical anesthetic (Proparacaine) and standard ROP documentation forms to screen for and treat ROP.

## Method for examining the fundus:

After the pediatric eye speculum and topical anesthesia with Proparacaine drops were used, an indirect ophthalmoscopy with a +20D lens was used to look at both funduses. The temporal retina received special attention and the results were recorded following the International Classification of Retinopathy of Prematurity (ICROP-3) rules for zone, stage and plus disease status.

## Distribution of treatment:

Patients who qualified for treatment were assigned to either intravitreal anti-VEGF therapy or laser photocoagulation, depending on the specifics of their disease and the doctor's judgment. Bevacizumab (0.625 mg/0.025 ml), ranibizumab (0.25 mg/0.025 ml) or aflibercept (0.5 mg/0.05 ml) were used as anti-VEGF agents and were given through a pars plicata injection. An 810nm diode laser was used for laser photocoagulation, which caused confluent burns to the avascular retina.

## Follow-up protocol:

Follow-up exams were set for one week, two weeks, one month, three months and six months after treatment. More visits were set up based on how active the disease was and how well the treatment worked. Follow-up assessments encompassed the evaluation of treatment efficacy, disease regression, necessity for retreatment and the emergence of complications.

## Outcome measures:

## The main outcome measures were:

[1] The rate of treatment success (complete disease regression)

[2] The need for retreatment or rescue therapy

[3] The time it took for the disease to regress

[4] Emergence of treatment complications

## Secondary outcome measures included:

[1] Visual development at 6 months corrected age

[2] Development of refractive error

- Structural outcomes (retinal detachment, vitreous hemorrhage)

- Adverse events that affect the whole body

## Classification of outcomes:

Treatment outcomes were categorized as favorable (complete regression of retinopathy of prematurity with a flat retina and ongoing physiological vascularization) or unfavorable (progression to Stage 4A/4B, Stage 5, development of a falciform fold or central media opacity hindering retinal evaluation).

## Collecting and managing data:

Standardized case record forms were employed for methodical data collection. All clinical findings, treatment specifics and subsequent observations were systematically documented and preserved in an electronic database format.

## Results & Discussion:

The study provided comprehensive findings regarding the demographic data, treatment allocation and outcomes of Retinopathy of Prematurity (ROP) cases. Table 1 shows the demographic information about the group. It included 1188 babies, with 61% being boys and 39% being girls. There were 511 preterm babies and 677 term babies. The average weight at birth was 2.3 kg and the average age at birth was 36.25 weeks. The average age for the first ROP screening was 38 days. Table 2 shows how treatments for Type 1 ROP were spread out. 67 babies got Intravitreal Anti-VEGF therapy and 10 babies got laser photocoagulation. Table 3 shows the results of babies who were treated with Anti-VEGF. It shows that 96.82% of their eyes had good results, while 3.17% needed more laser treatment. Table 4 shows the differences in results between Intravitreal Anti-VEGF and laser therapy. Anti-VEGF had 96.82% good results, while laser therapy had 100% good results. Table 5 talks about problems and extra treatments that happened after Anti-VEGF was given. It shows that 44 babies had ROP that got better on its own and didn't need any more treatment, while 19 babies needed more laser therapy. Table 6 shows how many babies were diagnosed with ROP and how they did with treatment. AROP was diagnosed in 66 babies and most of them did well with Anti-VEGF. Six babies with stage 4 and 5 ROP were sent for surgery and some of them needed extra laser therapy. Together, these tables give a full picture of the study's results on ROP treatment and its effects.

The current group of 1,188 infants had a male preponderation (61%) and a preponderation of term births (677 terms and 511 preterm), mean birth weight of 2.30kg and a mean gestational age of 36.25 weeks. The average postnatal age at the initial retinal -oedema- precipitating factor (ROP)-screening was 38 days. Out of 120 cases of infants who had Type 1 ROP, 67 had intravitreal anti-vascular endothelial growth factor (VEGF) injections and 10 cases had laser photocoagulation. The anatomical results of intravitreal anti-VEGF therapy were favourable in 96.82% of the eyes and 3.17% of the eyes needed additional treatment with laser. Laser photocoagulation in itself had a 100 per cent positive outcome rate. A small proportion of complications that occurred after anti-VEGF include cataract, retinal and tractionalmacular detachment, which highlights the importance of long-term surveillance. Aggressive posterior ROP (APROP) was seen in 5.5%obre ROP-most of preterm infants treated with anti-VEGF and regressed. Stage 4 and 5 ROP were not common and required vitreoretinal surgery and more laser treatment. These findings are consistent with those of Mintz-Hittner *et al.* [9], who showed a 96.6883% regression after intravitreal bevacizumab in stage 3+ ROP versus 89.5% after laser photocoagulation. On the same note, Stahl *et al.* [10] reported that intravitreal ranibizumab achieved 80% success rate compared to laser 66 and the structural complications were less in the anti-VEGF group. Roohipoor *et al.* [17] in contrast found similar rates of regression (95 percent vs. 97 percent) between bevacizumab and laser, yet found more retreatment rates following anti-VEGF. Our adjunctive laser rate (3.17% is comparable to the 4% of Dogra and Vinekar [8] in an Indian tertiary cohort. Krungkraipetch *et al.* (2026) [18] meta-analysis (15 studies, 1,784 eyes) confirms no overall difference in recurrence (RR 1.78, 95% CI 0.56-5.65) or retreatment (RR 1.80) between anti-VEGF and laser, though zone II anti-VEGF had higher recurrence (RR 3.42) requiring monitoring consistent with your low retreatment need. Hartnett and Stahl's (2023) [19] review highlights anti-VEGF's shift from laser for zone I but notes recurrence risks (7-50+ week’s post-treatment). A number of studies have emphasized the refractive benefits of the anti-VEGF. Hoppe *et al.* [20] found that there was less incidence of myopia at the age of 2 years in the group of infants receiving ranibizumab compared to those receiving laser. Similarly many studies reported that eyes treated with anti-VEGF agents are associated with lower levels of myopia than those treated with laser [20]. Studies differ on the dosages of agents and ages of infants when they are treated. On the other hand, McLoone *et al.* [7] reported the peripheral visual-field constriction following laser, which supports the use of anti-VEGF to maintain retinal functioning. Our cohort had a complication profile that was comparable to that of the complication profile described by Hwang *et al.* [21], who had a 2 -percent cataract rate and a 1.5 -percent detachment rate following bevacizumab. It is still difficult to manage APROP. Jang *et al.* [22] showed that anti-VEGF followed by laser regression was 100 percent with combination of bevacizumab and laser, which agrees with our strategy of anti-VEGF and then laser on demand. Similar to our surgical procedures in Stage 4/5 cases, Bahrani and Alhasseny [23] recommended early vitrectomy following anti-VEGF failure to maximize outcomes in advanced ROP.

## Conclusion:

Intravitreal anti -VEGF in this large cohort exhibited high regression with low adjunctive laser need whereas in comparison with laser, it preserved peripheral retina and limited myopic shift. Laser, though, demonstrated a somewhat better primary anatomic success rate, pointing out that each of the modalities has its unique structural and refractive benefits that can and should be selected and used based on ROP zone, stage and recurrence risk.

## Figures and Tables

**Table 1:** Demographic data of cohort

**Category**	**Total Babies (N=1188)**	**Male Babies (N=719)**	**Female Babies (N=469)**
Total Number of Babies	1188	719 (61%)	469 (39%)
Preterm Babies	511	-	-
Term Babies	677	-	-
Mean Birth Weight	2.3 kg	-	-
Mean Gestational Age	36.25 weeks	-	-
Age at First ROP Screening	38 days	-	-

**Table 2:** Treatment distribution for type 1 rop

**Treatment Type**	**Number of Babies**
Intravitreal Anti-VEGF	67
Laser	10

**Table 3:** Outcomes after intravitreal anti-VEGF

**Outcome**	**Number of Eyes**	**Percentage**
Favourable Outcome (Anti-VEGF)	88	96.82%
Anti-VEGF Followed by Laser	34	-
Non-Favourable Outcome (Anti-VEGF)	3	3.17%
Anti-VEGF Followed by Laser	1	-

**Table 4:** Outcome comparison between intravitreal anti-VEGF and laser

**Treatment Type**	**Favourable Outcome (%)**	**Non-Favourable Outcome (%)**
Intravitreal Anti-VEGF	96.82%	3.17%
Laser	100%	0%

**Table 5:** Complications and supplementary treatments after intravitreal anti-VEGF

**Outcome**	**Number of Babies**	**Details**
Regressing ROP and Mature Retina	44	No further intervention required
Supplement Laser Required	19	Babies needed additional laser therapy post-VEGF
Retinal Detachment & Cataract (with laser)	1	Zone 1 AROP, plus disease, developed cataract and retinal detachment
Retinal Detachment & Tractional Macular Detachment	1	Post Anti-VEGF followed by laser treatment

**Table 6:** Incidence of ROP and treatment outcomes in the cohort

**ROP Diagnosis Type**	**Total Babies Diagnosed**	**Treatment Given**	**Outcome**
AROP	66	Intravitreal Anti-VEGF	Majority had regressing ROP and mature retina
Type 1 ROP	83	Intravitreal Anti-VEGF + Laser	Regressing ROP with some requiring further laser
Stage 4 and 5 ROP	6	Vitreoretinal Surgery	Referred for further surgery, with some needing supplementary laser
